# How to Enhance Dental Implant Therapies and Definitive Restoration Outcomes to Reduce Complications and Improve Patient Well-Being

**DOI:** 10.3390/ma16103730

**Published:** 2023-05-15

**Authors:** Jordi Gargallo-Albiol

**Affiliations:** 1 Oral and Maxillofacial Surgery Department, UIC Barcelona-Universitat Internacional de Catalunya, 08195 Barcelona, Spain; jgargallo@uic.es; 2 Periodontics and Oral Medicine Department, School of Dentistry, University of Michigan, Ann-Arbor, MI 48109, USA

Dental implants have changed modern dentistry, providing a long-term, effective solution for tooth loss. Scientific evidence has clarified the factors associated with dental implant success, including the employed implant materials, implant macro- and micro- designs, placement techniques, and other patient-related factors such as bone quality and quantity. This evidence also comprises several recent studies that have contributed to a better understanding of the biomechanics of dental implants and improving tissue responses to the materials used for definitive implant restorations. The application of new technologies in the field of dental implantology, the use of new instruments, the development of new materials, and, as mentioned above, the criteria regarding the application of the different techniques and advances in new materials have facilitated the elucidation of new standards of success, a reduction in complications, and the improvement of patients’ well-being.

This Special Issue collects the most remarkable results of 15 recent investigations, constituting an open door with which to visualize the scientific advancements in implant dentistry.

Marginal bone loss (MBL) entails dental implant therapy monitoring, offering objective data regarding the present situation and its long-term evolution. Three of the studies included in this Special Issue evaluated the effects of implant positioning depth with respect to bone crests in relation to marginal bone loss. No significant differences in MBL were found in implants placed at crestal and sub-crestal levels [1] or by using a novel triangular-neck implant design (V3-Mis Dental Implant) placed at different implant depths by inexperienced post-graduate students [2]. It seems that inexperienced operators, such as postgraduate students in oral surgery and periodontology programs, have a low failure rate (comparable to that of experienced practitioners) when analyzing implant placement and posterior rehabilitation [3]. Thus, it can be concluded that modern dental implant treatment represents a highly predictable therapy regardless of whether it is executed by either experienced or non-experienced operators.

Moving on to the study of materials constituting potential alternatives to titanium in dental implants, one study explored the clinical and radiographic outcomes of zirconia implants (pure ceramic, Straumann), finding that one-piece zirconia implants seem to be a feasible option in patients with favorable bone anatomy, offering optimal clinical and radiographic outcomes after a mean follow-up period of 34.05 months (range 24–84). However, the authors indicated the necessity of further research involving prospective studies to evaluate these implants’ long-term performance, particularly in challenging situations such as bone deficiencies, immediate implant placement, or immediate-loading scenarios. Nevertheless, zirconia seems to be the best-positioned alternative to titanium [4].

With regard to bone reconstruction, it is clear that bone deficiencies no longer preclude the insertion of dental implants. Surgical techniques for rebuilding lost anatomical structures enable the recovery of functionality and the capacity to insert dental implants. Far from the mouth environment, one study showed that bone and cartilage autografts and synthetic grafts, combined with specific implants, represent good alternatives for orbital wall reconstruction [5].

Analyzing maxillary bone regeneration and different presentations of β-tricalcium phosphate (β-TCP) (synthetic grafts) on maxillary sinus floor elevation procedures, Mendes et al. 2022 [6] still observed superior results when using autogenous bone grafts. On the other hand, similar results have been found when comparing two different presentations of β-TCPs, indicating that the variation in the presentation of β-TCPs had minimal incidence in the final results regarding sinus floor elevation. Contrarily, when considering bone reconstruction in a less favorable scenario, such as a procedure concerning an area beyond the bony envelope, the use of a three-dimensional preformed titanium mesh (3D-PFTM) for peri-implant dehiscence defects situated in the anterior maxilla may achieve predictable hard tissue volume stability independently of the simultaneous use of a cross- or non-cross-linked collagen membrane [7]. Nevertheless, it is important to emphasize that the authors highlight the need to conduct more studies involving long-term results following functional loading before recommending this technique for routine clinical use.

An important step preceding the use of extensive bone regeneration techniques, which allow for predictable dental implant placement, consists of the clinical procedures that can prevent bone atrophy after tooth loss and, consequently, predict the need to apply more extensive bone reconstructive techniques. One of these approaches is the application of socket preservation techniques, which, when correctly applied, can successfully reduce volumetric bone ridge changes after tooth extraction. Concerning instances where immediate implant placement is not performed, independently of the reason for which this decision treatment approach was employed, a decision tree for alveolar ridge preservation procedure has been clearly provided in the study published in this Special Issue by Steigmann et al. 2022 [8], which serves to guide clinicians toward the most conservative and predictable treatment approach based on the remaining socket anatomical structures after extraction.

With regard to the biomechanics of dental implants and understanding the tissue responses to different bone ostectomy approaches and implant macro-designs, these parameters will undoubtedly influence the final outcome of implant therapy. One study published in this Special Issue has found that the bone tap osteotomy technique (involving a bone cavity prepared by modeling threads in accordance with the implant macro-design being inserted) exerted less stress on bone compared to conventional osteotomies (involving a bone cavity consisting of no modification, where neither the cortical crest region nor the bone-tap-inducing screw threads are involved) and even the countersink technique (involving a bone cavity prepared with a 1.2 mm deep and 0.5 mm excess diameter preparation on the coronal part of the bone ostectomy) [9]. These results intimate the importance of bone tapering, which is a surgical step that has been extensively performed in the past. Nevertheless, with the use of more aggressive implant macro-design in terms of self-tapering, involving conical designs to enhance implant primary stability, bone tapering is a surgical step that has decreased. Hence, bone stress is always greater when inserting implants into cortical bone than in cancellous bone, independently of the type of bone ostectomy performed, and the bone tap technique always helps to reduce the bone stress transferred to any kind of bone during implant insertion, and may once again play an important role in modern implantology.

Concerning the flexibility of the materials employed for the final implant-supported rehabilitations and the association with the stress transferred to the bone, the study by Medina-Galvez et al. [10] revealed that more flexible materials transfer less stress than stiffer materials such as metal and ceramic. When dynamic forces are applied to dental-implant-fixed restorations, these forces transfer differently to the bone depending on the type of rehabilitation material, for which higher transmission with ceramic restorations than composite restorations is observed. Additionally, the presence or absence of cortical bone also influences the transmission of force, presenting major force transmission when cortical bone is present. Thus, in the absence of cortical bone, such as in cases of peri-implantitis, bone regeneration, and immediate implant placement, the crown material to be employed for final rehabilitation must be carefully considered.

However, materials’ flexibility can influence abutment height modifications in relation to the different torques applied to the implant abutment. The distance from the implant platform to the top of the transepithelial abutment can be changed through the application of manual torque (10 Ncm) or mechanical torque (30 Ncm) [11].

Continuing the discussion of dental-implant-supported rehabilitation materials, it is possible to delve deeper into the micro-structure and analyze the powders to elaborate their zirconia and ceramic components in order to improve the powders’ biomedical applications for dentistry. The alkaline treatment of zirconia provides a better surface with improved “wetting” properties [12] favoring a better interaction between Ca-deficient hydroxyapatite with modified ZrO_2_ surfaces.

In line with the study of material properties but concerning the body surface of a dental implant, one study included in this Special Issue elucidated the incorporation of a double-acid-etched surface treatment (Avantblast^®^) that may enhance survival and success rates in comparison to the typically employed acid-etched surfaces. The study includes 10-year-post-loading data collected through a cohort clinical study [13].

Finally, the well-being and sensations perceived by patients are important inputs to consider when evaluating the success of dental implant therapy. An investigation collected in this Special Issue has found a significant decrease in tactile sensitivity with mandibular-implant-supported and maxillary-mucosa-supported prostheses in comparison to patients with natural dentition [14]. Although dental implant treatments have grown vastly and are a widely predictable solution in tooth loss scenarios, natural dentition still represents the gold-standard solution regarding functional and tactile patient perceptions. In consequence, it is not unusual that auto-transplanted teeth integrating the application of current 3D-planning advances have achieved new standards of success. According to the study published by Lucas-Taulé et al., the surgical repositioning of a tooth in another surgical site within the same individual (known as tooth autotransplantation) is a viable option before performing an implant therapy, offering high survival and success rates regardless of the apical condition [15].

In summary, the studies published and recompiled in this Special Issue demonstrate the continuous significant advancements made in implant dentistry, showing new scientific evidence on new material properties, application techniques, and technologies for enhancing implant placement therapies and definitive restoration outcomes. Further research is still needed to assess the long-term performance of all these advancements in different clinical situations, but the results obtained thus far are promising. Implant dentistry continues to evolve, and it will undoubtedly become better and more effective in the near future, benefiting both patients and dental professionals.

## References

[1] Palacios-Garzón N., Mauri-Obradors E., Ayuso-Montero R., Velasco-Ortega E., Anglada-Cantarell J.M., López-López J. (2022). Marginal Bone Loss in Internal Conical Connection Implants Placed at the Crestal and Subcrestal Levels before Prosthetic Loading: A Randomized Clinical Study. Materials.

[2] Giralt-Hernando M., Ragucci G.M., Cantó-Naves O., Valls-Ontañón A., Hernández-Alfaro F. (2022). Covariates Relating to Implant Failure and Marginal Bone Loss of a Novel Triangular Neck-Implant Placed by Post-Graduate Students: A 1-Year Prospective Cohort Study. Materials.

[3] Ragucci G.M., Giralt-Hernando M., Méndez-Manjón I., Cantó-Navés O., Hernández-Alfaro F. (2020). Factors Affecting Implant Failure and Marginal Bone Loss of Implants Placed by Post-Graduate Students: A 1-Year Prospective Cohort Study. Materials.

[4] Gargallo-Albiol J., Böhm K., Wang H.-L. (2022). Clinical and Radiographic Outcomes of Zirconia Dental Implants&mdash;A Clinical Case Series Study. Materials.

[5] Vasile V.A., Istrate S., Iancu R.C., Piticescu R.M., Cursaru L.M., Schmetterer L., Garhöfer G., Cherecheanu A.P. (2022). Biocompatible Materials for Orbital Wall Reconstruction—An Overview. Materials.

[6] Mendes B.C., Pereira R.S., Mourão C.F.A.B., Montemezzi P., Santos A.M.S., Moreno J.M.L., Okamoto R., Hochuli-Vieira E. (2022). Evaluation of Two Beta-Tricalcium Phosphates with Different Particle Dimensions in Human Maxillary Sinus Floor Elevation: A Prospective, Randomized Clinical Trial. Materials.

[7] Lee S.-R., Jang T.-S., Seo C.-S., Choi I.-O., Lee W.-P. (2021). Hard Tissue Volume Stability Effect beyond the Bony Envelope of a Three-Dimensional Preformed Titanium Mesh with Two Different Collagen Barrier Membranes on Peri-Implant Dehiscence Defects in the Anterior Maxilla: A Randomized Clinical Trial. Materials.

[8] Steigmann L., Di Gianfilippo R., Steigmann M., Wang H.-L. (2022). Classification Based on Extraction Socket Buccal Bone Morphology and Related Treatment Decision Tree. Materials.

[9] Mahendra J., Chand Y.B., Mahendra L., Fageeh H.N., Fageeh H.I., Ibraheem W., Alzahrani K.M., Alqahtani N.M., Alahmari N.M., Almagbol M. (2021). Evaluation of Stress Distribution during Insertion of Tapered Dental Implants in Various Osteotomy Techniques: Three-Dimensional Finite Element Study. Materials.

[10] Medina-Galvez R., Cantó-Navés O., Marimon X., Cerrolaza M., Ferrer M., Cabratosa-Termes J. (2021). Bone Stress Evaluation with and without Cortical Bone Using Several Dental Restorative Materials Subjected to Impact Load: A Fully 3D Transient Finite-Element Study. Materials.

[11] Cordeiro B.Q.S., Mourão C.F.A.B., Carvalho W.R., Fonseca E.M., Montemezzi P., Javid K., Martins C.C., Quinelato V., Moreno M.D., Casado P.L. (2021). Vertical Discrepancy in Height of Morse Cone Abutments Submitted to Different Torque Forces. Materials.

[12] Nakonieczny D.S., Martynková G.S., Hundáková M., Kratošová G., Holešová S., Kupková J., Pazourková L., Majewska J. (2022). Alkali-Treated Alumina and Zirconia Powders Decorated with Hydroxyapatite for Prospective Biomedical Applications. Materials.

[13] Santos Marino J., Cortés-Bretón Brinkmann J., García-Gil I., Martínez-Rodríguez N., Fraile J.F., Barona Dorado C., Martínez-González J.M. (2021). Clinical Evaluation of Dental Implants with a Double Acid-Etched Surface Treatment: A Cohort Observational Study with Up to 10-Year Follow-Up. Materials.

[14] Moraes N., Moraes E., Anastacio T., Silva L., Machado A., Schoichet J., Alto R.M., Mello-Machado R., Cardarelli A., Mourão C.F.A.B. (2021). Active Tactile Sensibility of Brånemark Protocol Prostheses: A Case–Control Clinical Study. Materials.

[15] Lucas-Taulé E., Bofarull-Ballús A., Llaquet M., Mercade M., Hernández-Alfaro F., Gargallo-Albiol J. (2022). Does Root Development Status Affect the Outcome of Tooth Autotransplantation? A Systematic Review and Meta-Analysis. Materials.

